# Implementing structured team debriefing using a Black Box in the operating room: surveying team satisfaction

**DOI:** 10.1007/s00464-020-07526-3

**Published:** 2020-04-06

**Authors:** A. S. H. M. van Dalen, M. Jansen, M. van Haperen, S. van Dieren, C. J. Buskens, E. J. M. Nieveen van Dijkum, W. A. Bemelman, T. P. Grantcharov, M. P. Schijven

**Affiliations:** 1 Department of Surgery, Amsterdam UMC, University of Amsterdam, Amsterdam, The Netherlands; 2 Clinical Research Unit, Amsterdam UMC, University of Amsterdam, Amsterdam, The Netherlands; 3 Department of Anaesthesiology, Amsterdam UMC, University of Amsterdam, Amsterdam, The Netherlands; 4 International Centre for Surgical Safety, St Michael’s Hospital, Toronto, Canada; 5 Department of Surgery, Amsterdam Gastroenterology and Metabolism, Amsterdam UMC, University of Amsterdam, Amsterdam, The Netherlands

**Keywords:** Surgical safety, Medical data recorder, Black Box, Team debriefing, Training, Operating room

## Abstract

**Background:**

Surgical safety may be improved using a medical data recorder (MDR) for the purpose of postoperative team debriefing. It provides the team in the operating room (OR) with the opportunity to look back upon their joint performance objectively to discuss and learn from suboptimal situations or possible adverse events. The aim of this study was to investigate the satisfaction of the OR team using an MDR, the OR Black Box®, in the OR as a tool providing output for structured team debriefing.

**Methods:**

In this longitudinal survey study, 35 gastro-intestinal laparoscopic operations were recorded using the OR Black Box® and the output was subsequently debriefed with the operating team. Prior to study, a privacy impact assessment was conducted to ensure alignment with applicable legal and regulatory requirements. A structured debrief model and an OR Back Box® performance report was developed. A standardized survey was used to measure participant’s satisfaction with the team debriefing, the debrief model used and the performance report. Factor analysis was performed to assess the questionnaire’s quality and identified contributing satisfaction factors. Multivariable analysis was performed to identify variables associated with participants’ opinions.

**Results:**

In total, 81 team members of various disciplines in the OR participated, comprising 35 laparoscopic procedures. Mean satisfaction with the OR Black Box® performance report and team debriefing was high for all 3 identified independent satisfaction factors. Of all participants, 98% recommend using the OR Black Box® and the outcome report in team debriefing.

**Conclusion:**

The use of an MDR in the OR for the purpose of team debriefing is considered to be both beneficial and important. Team debriefing using the OR Black Box® outcome report is highly recommended by 98% of team members participating.

**Electronic supplementary material:**

The online version of this article (10.1007/s00464-020-07526-3) contains supplementary material, which is available to authorized users.

Despite various efforts aiming to improve surgical safety, the incidence of surgical adverse events remains high to date [1–3]. Studies have estimated one-third of surgical adverse events to be potentially preventable [1, 2, 4, 5]. Adverse events are usually not the result of individual failure, but the consequence of an uninterrupted chain of events and decisions, spanning multiple phases of surgical care. An important number of these adverse events occur within the operating room (OR) and are most often unnoticed by the team [2, 6, 7]. Therefore, a suggested approach towards error reduction could focus on finding and implementing mechanisms to facilitate the awareness of such unnoticed events [8]. Subsequently, steps should be undertaken to acknowledge, analyse and understand common error-event patterns [7, 8]. Several studies have highlighted the importance of non-technical skills in the OR to avoid error. Skills associated with error reduction or prevention are teamwork, situational awareness and communication [9–11]. Therefore, interventions to improve surgical quality and safety should involve all members of the operating team [11–13].

A Medical Data Recorder (MDR) is similar to a system better known in aviation as a ‘Black Box’ or a ‘Flight Data Recorder’. It may have the potential to look back upon joint performance jointly to improve quality and safety in the OR. The outcome of using an MDR may be used for purposes of multidisciplinary debriefing in a privacy-protected environment if it is well constructed for this purpose. This may provide surgical teams with the opportunity to assess unnoticed events and look back upon their actual performance to learn and improve. Hence, it may avoid future adverse events that possibly compromise surgical safety.

Despite aforementioned insights and currently available technology, reported surgical safety improvement initiatives using an MDR are still limited. Moreover, an actual multidisciplinary debriefing culture for teams performing surgery is lacking [14–17].

The aim of this study was to investigate the participants’ satisfaction with an MDR, the OR Black Box® and its subsequent performance report used as a tool for structured postoperative multidisciplinary debriefing [18].

## Methods

### Participants, privacy and surgical case selection

To ensure the privacy of all participants, the research protocol was checked to be compliant with applicable privacy, legal and regulatory requirements by conducting an official Privacy Impact Assessment (PIA) [19]. Legal guidelines were explored before set-up of study [19]. This study was approved by the Hospital Directorate and Works Council (staff representation). An institutional review board (IRB) approval did hence not have to be obtained [19].

The research coordinators (AvD and MS) gave several oral presentations at the different clinical departments involved in the OR to inform all participants about the Transparency in the Operating Room (TOPPER) trial. The objectives and methods were explained, questions were answered and they were asked to give their written informed consent prior to participation.

From February 2017 until January 2018, consecutive elective gastro-intestinal laparoscopy cases were recorded using the OR Black Box® (Surgical Safety Technologies Inc., Toronto, Canada). The standardized questionnaire was tested for its adequacy and measured the operating team’s satisfaction, using factor analysis for optimal assessment of underlying constructs. Patients were pre-operatively informed about the study and asked whether they would have any objections to be operated in an OR where an MDR was being used (“opt-out” option) [19].

### Operating room set-up

**Fig. 1:**
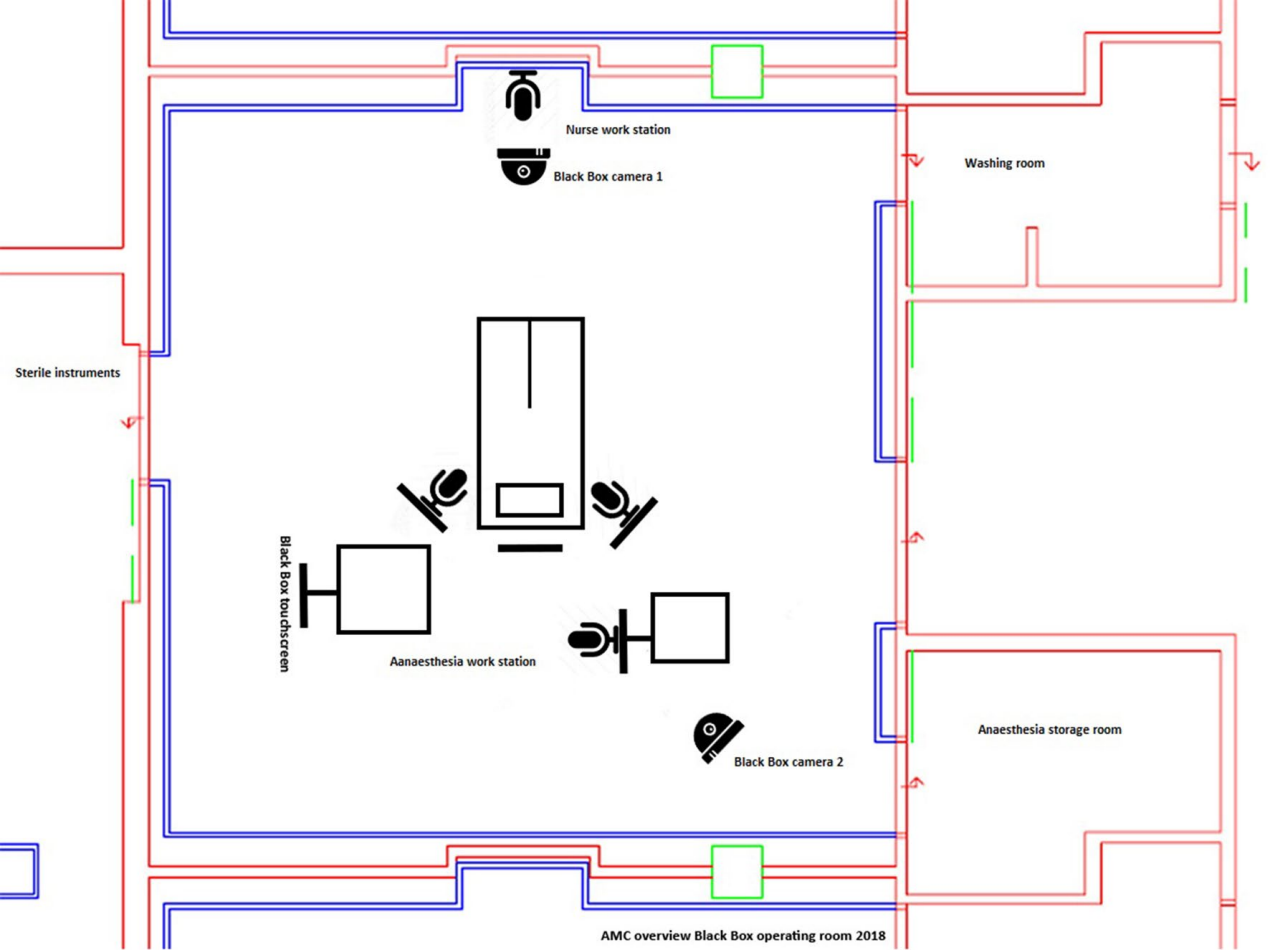
Overview of the operating theatre including position of the ceiling-mounted cameras and OR Black Box® microphones, attached to the operating theatre monitors

The OR Black Box® is an MDR that was installed in the ‘ENDOALPHA’ operating suite (Olympus Europa SE & Co. KG, Hamburg) in the Amsterdam University Medical Centres, location AMC [18, 20]. This recorder is able to capture a multitude of data streams in perfect synchronization. Figure 1 depicts the OR theatre set-up, including the position of the cameras, microphones and OR Black Box® touchscreen.

Cases were recorded between the time-out and sign-out time stamp of the surgical procedure, according to most recent SURPASS (Surgical Safety Checklist) guidelines, with the consented patient fully draped to optimally ensure the consented patient’s privacy [19, 21, 22]. Patient parameters were recorded and retrieved in real time via the anaesthesia monitor. All captured data were collected upon generation by the OR Black Box® encoder, stripped from personal identifiers and subsequently synchronized. Immediately following, the dataset was securely encrypted by the OR Black Box® system before it was transmitted to the Canadian contractor. This was done with secure Virtual Private Network technology (VPN) using a system push command upon action of the study investigator, immediately after procedural sign-out.

### Construction of the Black Box performance report

The OR Black Box® dataset was decrypted and analysed partly using software algorithms by the contractor, the Surgical Safety Centre (Canada, Toronto). Subsequently, deep-learning algorithms flagged ‘near miss’ events in the dataset, and events were ‘tagged’ when they were considered to be relevant. Following, the dataset was analysed by the OR Black Box® analysis team (a specialized trained team of surgeons and human factors specialists) in full to double-check for fault-positive, negative and inappropriate placed flags of the learning algorithms in order to avoid faulty analysis. Since the software and analysing team uses English as primary language, the team was asked to speak English during the recording of the surgical cases. Study participants were told that they could always revert back to Dutch, if necessary. Yet, the debriefings were done in Dutch. As the contractor of the MDR resides in Canada, the Canadian analysis team was briefed about local standard operating procedures before start of study, by all the participating surgeons. The analysis was based on well-known, scientifically validated rating scales that can be found in literature, such as the System Engineering Initiative for Patient Safety (SEIPS) model of work system and patient safety, the Non-Technical Skills for Surgeons (NOTSS), The Scrub Practitioners' List of Intraoperative Non-Technical Skills (SPLINTS) system and the Disruptions in Surgery Index (DISI) [23–26]. This original ‘tagged performance report’ was considered to be too lengthy and granular for feasible debriefing the operating team, hence it was further translated into a graphical summarized performance report.

**Fig. 2:**
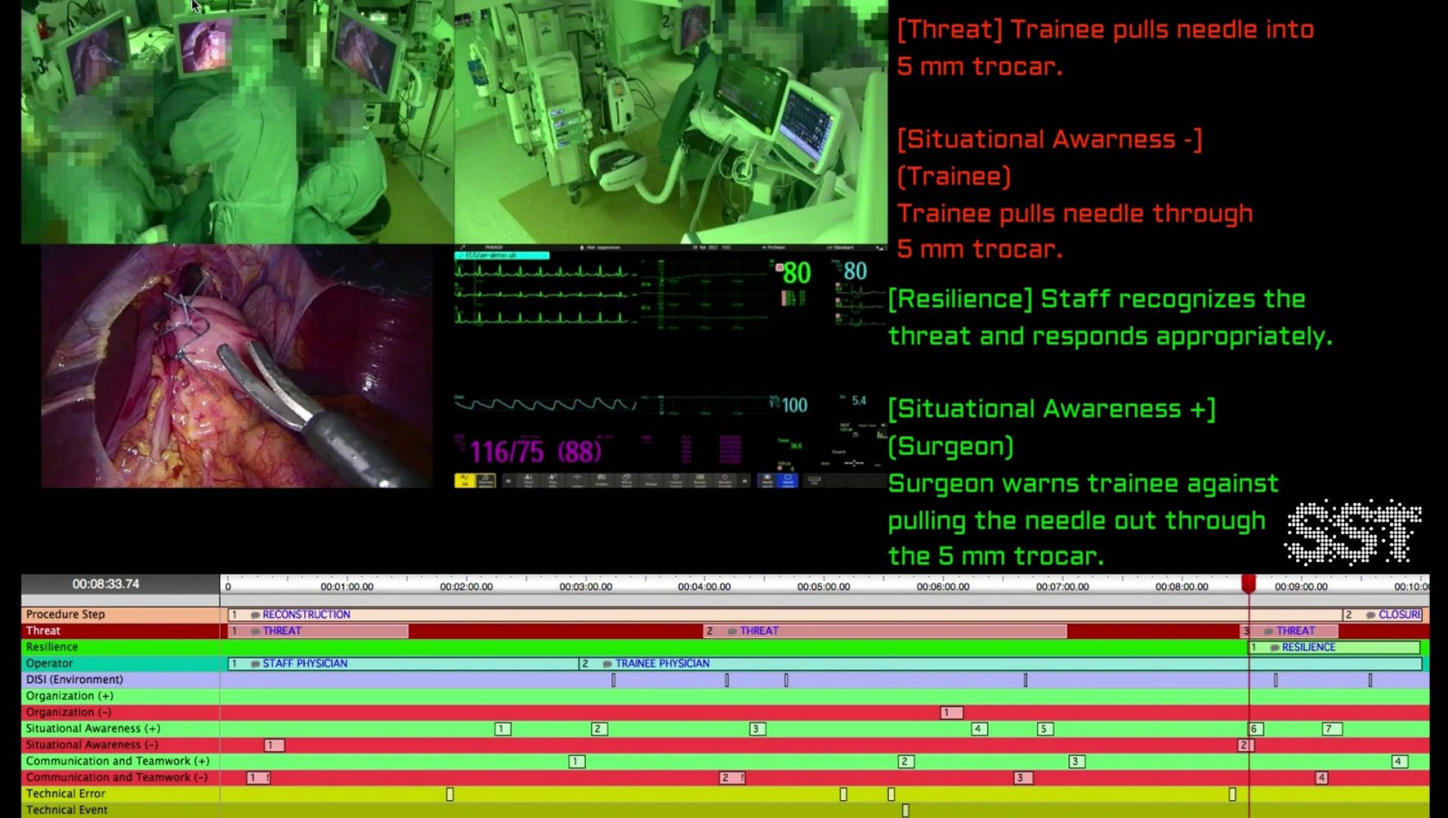
Example of the OR Black Box® performance report, including video clip, used in the postoperative team debriefings

This graphical performance report model compromised a summarized ‘video clip’ of about 10 min. Figure 2 shows an example of the OR Black Box® performance report. The video clip included the 2 overview camera’s, the anaesthesia monitor and laparoscopic camera as depicted in Fig. 1 and 2. The structured feedback from the OR Black Box® analysis team (Toronto, Canada) was added to the summarized ‘video clip’ in annotations, including all relevant positive (green line) and negative events (red line) of the particular case. As shown in Fig. 2, the timeline of the procedure and video clip is visualized in the lower part of the report. The green and red lines represent the positive and negative rated human factor events. The green or red squares within these lines represent a specific safety threat or resilience support event for which written feedback is provided in the right upper part of the report. These events were discussed during the team debriefing.

All personally identifiable information was stripped from the performance report (faces are blurred, voices were altered and patient data were removed). The original OR Black Box® data were analysed within 48 h and the resulting outcome report was securely sent back to the project coordinators (AvD and MS), to be used for the debriefings only.

### Team debriefing

The procedures were debriefed in a standardized way with the help of a, by the authors (AvD and MvH), developed debrief model to be used with the OR Black Box® output. The debriefing methods are presented in another study and were based on insights derived from literature review [27–29]. Represented in the model are the following categories: environment, organization, situational awareness and communication & teamwork. The debriefing sessions were done in Dutch. The debriefings were led by an “independent moderator” (a professor of psychiatry) to structure the debriefing process optimally, by guiding the process and providing feedback as neutral as possible whilst maintain a trustful relationship within the team [28, 29].

### Questionnaire and statistical analyses

All survey data collection and statistical analyses were executed by the authors at our academic medical centre (AvD, and SvD) to adjudicate possible conflicts of interest. The founder and equity holder (TG) of Surgical Safety Technologies (SST) Inc., Toronto, Canada was involved in the co-development and delivery of the structured performance outcome reports, but not in set-up nor outcome analysis of study.

Following the TOPPER-trial team debriefing sessions, participants completed a standardized questionnaire surveying user satisfaction regarding the performance report and OR Black Box® as a tool for team debriefing. The original questionnaire is written in Dutch and can be found in the Appendix. As the debriefing was also done in Dutch and the questionnaire was analysed by the Dutch study coordinator (AvD, SvD), it was not translated to English.

Exploratory factor analysis of the questionnaire was used to measure the satisfaction of the users. This included a principal-axis factor analysis which was conducted on the 23 items (10-point Likert scale questions) with oblique rotation. The Kaiser–Meyer–Olkin (KMO) and Bartlett’s test was used to verify the sample size adequacy of the completed satisfaction questionnaires. The correlation matrix and anti-image matrix (values < 0.5) were used to decide which questions had to be removed, because these questions correlated too highly (> 0.9) or poorly (< 0.2). The questions clustered in the satisfaction factors were tested for reliability by the Cronbach’s α test (> 0.7) [30].

Linear regression analysis was used to determine whether independent covariates were significantly correlated with the, in the factor analysis identified, different satisfaction factors. Covariates with a threshold *p* value of 0.20 were entered in the multivariable linear regression model. Multivariable regression analysis was performed to estimate differences in variables associated with the selected satisfaction factors. The multivariable regression model was created using a backward stepwise fashion. Covariates in the multivariable regression model with a threshold *p* value of 0.05 were considered to be significantly associated with the outcome variable. The *B* values with 95% confidence intervals (CI) were presented. All statistical analyses were conducted using SPSS statistics 24.0 for Windows.

## Results

In total, 35 surgical procedures were recorded of which 18 were laparoscopic fundoplications, 6 laparoscopic diaphragmatic hernia repairs, 3 elective laparoscopic appendectomies, 3 laparoscopic subtotal colectomies, 2 laparoscopic unilateral adrenalectomies, 2 laparoscopic bilateral adrenalectomies and 1 laparoscopic sigmoid resection. In these cases, 4 surgeons, 2 surgical fellows, 12 surgical residents, 6 anaesthesiologists, 5 anaesthesiology residents, 9 anaesthesiology nurses, 27 theatre nurses and 16 medical interns participated (*N* = 81). The baseline characteristics of participants are presented in Table 1.

**Table 1:** Baseline characteristics

Role in the operating theatre	Age (median)	Gender (*N* per total cases)	Years working at this hospital (median)	Times participated in Black Box debriefing (*N* per total cases)	Optimal length of debriefing (minutes, mean)	Would you recommend participating in a Black Box team debriefing to your colleagues? (*N*, yes vs. no)
Primary surgeon	47.0 (IQR 1.0)	31 female 2 male	8.0 (IQR 1.0)	5 (first time) 8 (1–5 times) 5 (6–10 times) 14 (> 10 times)	27.1 (SD 5.7)	33 yes 0 no
Assisting surgeon	33.0 (IQR 6.0)	13 female 6 male	1.0 (IQR 4.5)	8 (first time) 11 (1–5 times) 0 (> 5 times)	20.7 (SD 8.3)	18 yes 1 no
Anaesthesiologist	41.0 (IQR 13)	8 female 12 male	7.0 (IQR 6.0)	9 (first time) 11 (1–5 times) 1 (6–10 times) 0 (> 10 times)	32.6 (SD 10.7)	18 yes 2 no
Anaesthesiology nurse	31.0 (IQR 26)	7 female 14 male	6.0 (IQR 3.5)	10 (first time) 11 (1–5 times) 0 (> 5 times)	33.9 (SD 14.9)	21 yes 0 no
Scrub nurse	29.0 (IQR 32)	18 female 1 male	3.8 (IQR 4.3)	5 (first time) 13 (1–5 times) 0 (> 5 times)	32.2 (SD 9.9)	18 yes 0 no
Circulating nurse	42.5 (IQR 18)	22 female 0 male	5.0 (IQR 17.0)	8 (first time) 13 (1–5 times) 0 (6–10 times) 1 (> 10 times)	40.5 (SD 11.5)	22 yes 0 no
Medical intern	25.0 (IQR 1.0)	11 female 7 male	–	10 (first time) 8 (1–5 times) 0 (> 5 times)	27.5 (SD 13.2)	18 yes 0 no

The debriefings took place approximately 14 working days (median, IQR 41) after the recorded procedure. On average, 4 (out of 7–8) team members (median, IQR 3) attended their team debriefing.

In total, 151 questionnaires were completed. The mean score on the question: “How important do you feel it is to be able to structurally debrief surgical procedures with the entire team” was 8.44 (SD 1.2, 10-point Likert scale).

### Factor analysis of the satisfaction questionnaire

The twenty-three questions, answered on a 10-point Likert scale, were evaluated in the factor analysis. The mean scores of each question are presented in Table 2. Mean scores of the questions demonstrated that the team members considered structured team debriefing to be important, useful, and educational.

**Table 2:** Overall mean scores per question of the standardized post-debriefing questionnaire and their corresponding factor(s)

Question	Overall mean score (10-point Likert scale, *N* = 151)	Factor loadings after rotation for Factor 1	Factor loadings after rotation for Factor 2	Factor loadings after rotation for Factor 3^a^
0. How important do you find it to be able to structurally debrief surgical procedures with the entire team?	8.4 (SD 1.2)	0.511	–	− 0.332
1. How would you rate today’s debriefing?	7.8 (SD 1.4)	0.803	–	–
*2.* How well met the covered topics with the predetermined goals of this team debriefing?	7.8 (SD 1.4)	0.339	0.611	–
3. How well-suited were the room and facilities for this debriefing?	8.5 (SD 1.1)	–	–	–
4. How well was this debriefing organized?	8.1 (SD 1.4)	–	–	–
5. Was content of the performance report useful for you?	7.8 (SD 1.6)	0.699	–	-–
6. Do you think the content of the performance report was useful for your team members?	8.2 (SD 1.3)	0.538	–	–
7. Do you estimate this debriefing to be of value to increase your own *situational awareness?*	8.4 (SD 1.2)	0.389	–	− 0.396
8. Do you estimate this debriefing to be of value to increase the *situational awareness* of operating teams, in general?	8.5 (SD 1.1)	–	–	− 0.698
9. Do you think participating in the Black Box debriefings will help you to communicate (even) better with your colleagues in the operating room?	8.6 (SD 1.1)	–	–	− 0.819
10. Do you think that participating in Black Box debriefings is of value for operating teams to better communicate with each other in the operating room?	8.5 (SD1.1)	–	–	− 0.887
11. Do you think that participating in Black Box debriefings is of value to be able to improve future teamwork in the operating theatre?	8.5 (SD 1.1)	–	–	− 0.801
12. Do you think that the OR Black Box® is a valuable instrument to enhance patient safety?	8.8 (SD 6.4)	–	–	− 0.555
13. Was this debriefing educational?	8.2 (SD 1.4)	0.846	–	–
14A. In case you learned something from this debriefing, to what extent do you expect it to be applicable in future surgical procedures?	8.7 (SD 5.7)	0.573	–	–
14B. In case you learned something from this debriefing, how motivated are you to practice in future surgical procedures?	9.96 (SD 9.7)	–	0.383	–
15. Did you find this debriefing to be useful?	8.1 (SD 1.5)	0.791	–	–
16. How well did this debriefing meet your expectations?	8.0 (SD 1.3)	0.743	–	–
17. Did you find the time you spent on attending this debriefing well spent?	8.2 (SD 1.3)	0.706	–	–
18. What is the ideal length of a team debriefing according to you? (minutes)	30.6 (SD 11.9)	–	–	–
19A. Of how much value would it be for you, to be able to choose which moments are being debriefed with the help of the anonymous video clips yourself?	7.5 (SD 1.9)	–	–	–
19B. Of how much value would it be for you to be able to get access to the performance report and/or anonymous video clips personally, after the Black Box procedure?	7.8 (SD 1.5)	–	0.655	–
20. How valuable did you find the anonymous video clips as part of the performance report?	8.4 (SD 1.1)	–	0.661	− 0.318
21. Do you find that, if available, it should be possible to use the OR Black Box® when the operating team wants to debrief a particular surgical procedure? (yes, no)	148 (98.7%, *N* = 150)	–	–	–
22. Would you recommend participating in a OR Black Box® team debriefing to your colleagues? (yes, no)	148 (98.0%, *N* = 151)	–	–	–
23. Did you miss something in the performance report? (yes, no)	25 (16.6%, *N* = 151)	–	–	–
24. Did you miss something in de briefing/method of debriefing with the performance report (including video clip)? (yes, no)	27 (17.9%, *N* = 151)	–	–	–
25. How satisfied are you with the use of the performance report (including video clips) as an instrument for structured operating team debriefing?	8.2 (SD 1.1)	–	0.624	–

^a^When all factor loadings in 1 factor are negative, they may be considered positive

The team members had a mean score of 8.2 (SD 1.1, 10-point Likert scale) regarding satisfaction with the use of the performance report (including video clip) as instrument for a structured operating team debriefing. Question 4 had a very low inter-correlation with question 14b and 20 (< 0.2) hence had to be excluded from the analysis (see Online Appendix)*.* An increase in Cronbach’s α to 0.851 was achieved by eliminating question 19b (factor 2). After exclusion of question 4 and 19b, a high KMO value of 0.937 and a significant Bartlett’s test (*p* value < 0.0001) confirmed that the questionnaire sample was indeed of adequate size for the analysis [31].

**Fig. 3:**
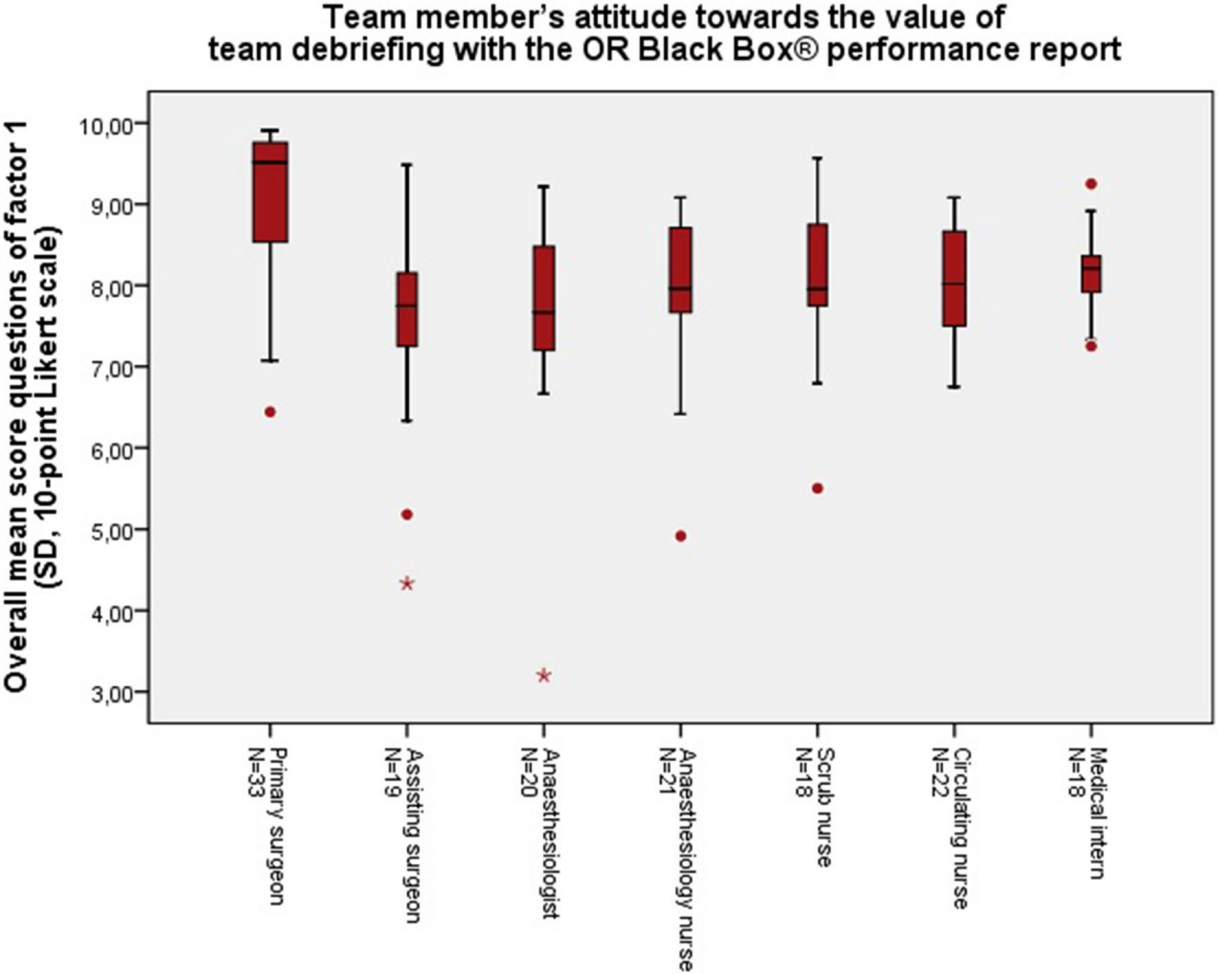
Total mean scores of the questions (Q0, Q1, Q2, Q5, Q6, Q7, Q13, Q14a, Q14b, Q15, Q16, Q17) included in *factor 1* representing the team member’s attitude towards the value of team debriefing with the OR Black Box®, per role in the operating theatre

**Fig. 4:**
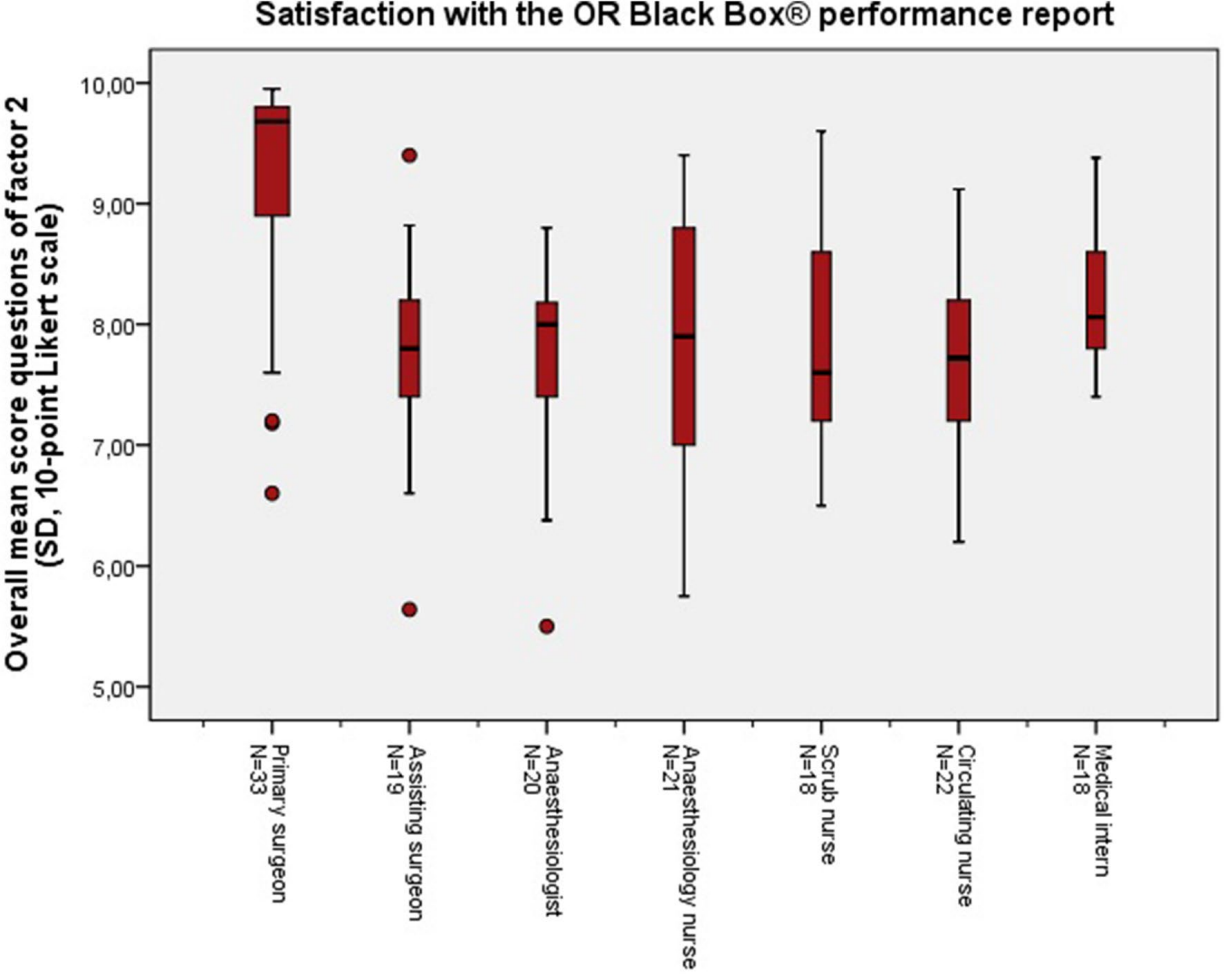
Total mean scores of the questions (Q2, A14b, Q19b, Q20, Q25) included in *factor 2* representing satisfaction with the OR Black Box® performance report, per role in the operating theatre

**Fig. 5:**
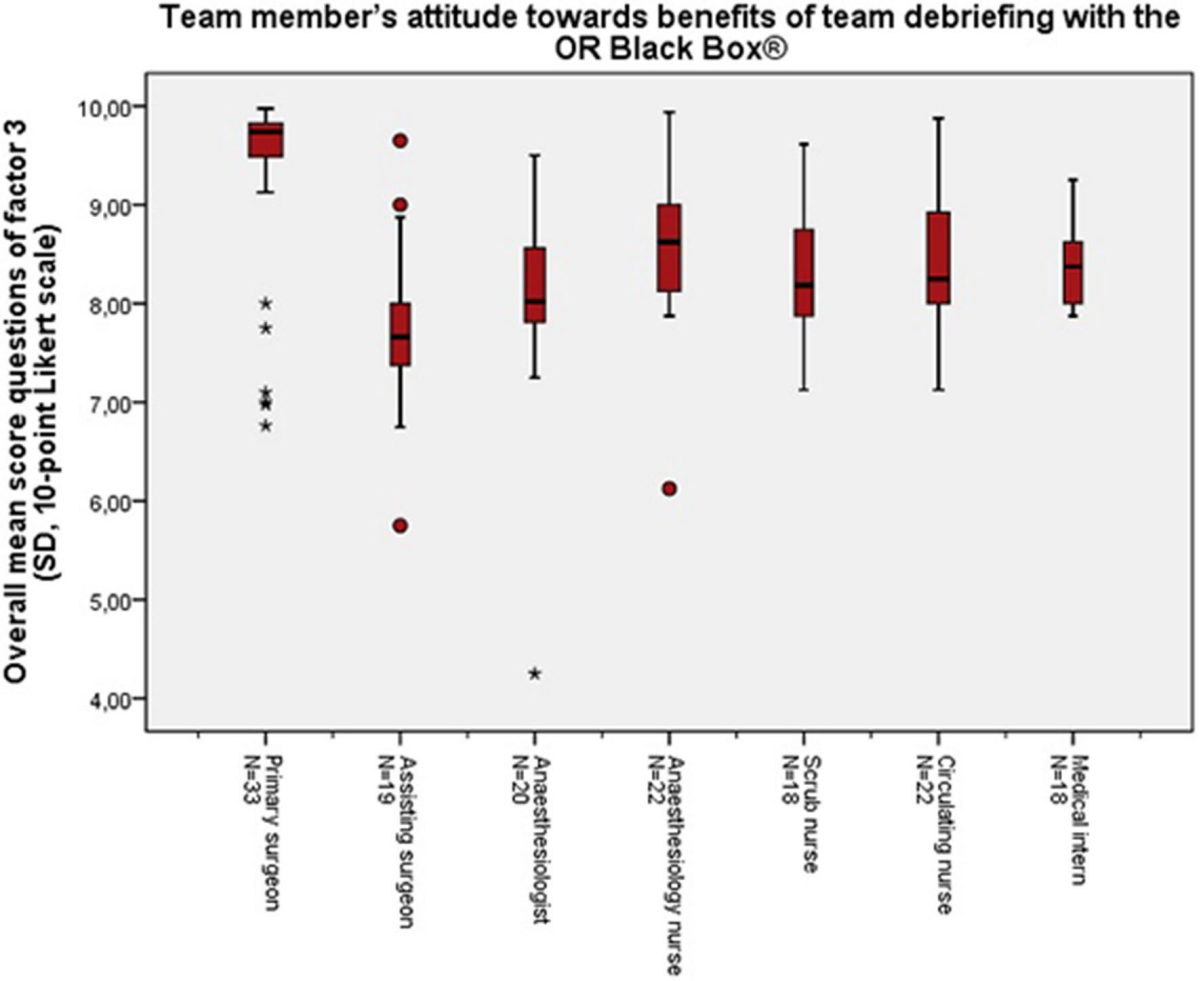
Total mean scores of the questions (Q0, Q7, Q8, Q9, Q10, Q11, Q12, Q20) included in *factor 3* representing the team member’s attitude towards benefits of team debriefing with the OR Black Box®, per role in the operating theatre

Resulting from the factor analysis, some questions clustered on three separate factors. These factors met the Kaiser’s criterion of 1 and in combination these 3 factors explained 64.9% of the variance (see Online Appendix). Factor 1 represents the team member’s attitude towards the “value of team debriefing with the OR Black Box® performance report”, i.e. whether it was useful and educational. Factor 2 represents the team member’s satisfaction with the use of the OR Black Box® performance report as instrument for a structured team debriefing. Factor 3 represents team member’s attitude towards the “benefits of team debriefing” with the OR Black Box®, i.e. the ability of the debriefings to improve the team’s communication, situational awareness and teamwork skills, and patient safety. Table 2 shows the factor loadings, per question (pattern matrix is attached in the appendix). The factor loadings demonstrate which questions clustered to factor 1, 2 or 3, respectively, and how much value they added to their factor. Figures 3, 4 and 5 show the overall mean scores, per role in the OR, of the questions included in factor 1, 2 or 3, respectively.

The multivariable linear regression, correcting for all potential confounders (simple linear regression table in the Online Appendix), showed that the primary surgeon was significantly more satisfied concerning all 3 satisfaction factors, compared to the other team members. Number of previously attended Black Box team debriefings was significantly associated with a higher satisfaction score for all 3 satisfaction factors (*Beta* coefficient = 0.29, 95%CI 0.09–0.49, *Beta* coefficient = 0.414, 95%CI 0.25–0.57, *Beta* coefficient = 0.422, 95%CI 0.59–0.26). Number of team members attending the team debriefing and number of work days between the procedure and debriefing were not significantly associated with the satisfaction scores. Total number of events reported in the performance report feedback was negatively associated with satisfaction factor 1 (*Beta* coefficient = − 0.013, 95%CI − 0.02 to − 0.002). Results of the multivariable linear regression analyses are presented in Table 3.

**Table 3:** Multivariable linear regression models for the 3 factors

Variables	Factor 1 Attitude towards value of team debriefing with the OR Black Box®		Factor 2 Satisfaction with the OR Black Box® performance report		Factor 3* Attitude towards benefits of team debriefing with the OR Black Box®	
Surgical procedure (upper-GI vs. adrenal, vs. colorectal)	–	–	–	–	–	–
Role in the OR (ref = main surgeon) assisting surgeon anaesthesiology (including anaesthesia-nurse) OR nurses (SN & CN)	*B* = − 0.652 *B* = − 0.329	95%CI − 1.13 to − 0.18 95%CI − 0.71 to 0.05	*B* = − 0.659 *B* = − 0.524 *B* = − 0.653	95%CI − 1.09 to − 0.23 95%CI − 1.0 to − 0.30 95%CI − 1.0 to − 0.30	*B* = − 0.842 – –	95%CI − 0.43 to − 1.26
Age	*B* = 0.016	95%CI − 0.001 to 0.034	–	–	–	
Sex	–	–	–	–	–	
Years working at the Amsterdam UMC	–	–	–	–	–	
Number of previously attended Black Box debriefings (first time, 1–5 times, 6–10 times, > 10 times)	*B* = 0.29	95%CI 0.09–0.49	*B* = 0.414	95%CI 0.25–0.57	*B* = 0.422	95%CI 0.59–0.26
Number of team members attending the debriefing	–	–	–	–	–	–
Number of work days between procedure and debriefing	–	–	–	–	–	–
Performance report feedback						
Total number of all (positive and negative) events in performance report	*B* = − 0.013	95%CI − 0.02 to − 0.002	–	–	–	–

^a^All values in this factor pattern matrix were negative, therefore negative values in the model may be considered positive and positive values may be considered negative

*B* The beta coefficient, which is the degree of change in the factor for every 1 unit of change in the predictor variable

## Discussion

This study focuses on the satisfaction of the OR team with the use of a new monitoring system, the OR Black Box®, and its subsequent output used in team debriefing. This outcome was chosen because for people working in the OR it is vital to feel comfortable and secure, in order to be able to adopt such an innovative system. The team has to be satisfied with a system that ‘watches’ and ‘judges’ them. Only then, a quest to learn from unnoticed or differently perceived errors may take place [32]. Overall, satisfaction of the surgical team with the use of the OR Black Box® and corresponding outcome performance report for postoperative structured team debriefing was very high. Ninety-eight percent of participants would recommend postoperative multidisciplinary debriefing with the use of the OR Black Box® derived output to their colleagues. Although team debriefing is not yet common practice in most hospitals, participating surgical team members have considered structured team debriefing to be important, useful, and educational [17, 33–37]. These results show that number of previously attended team debriefings is positively associated with user satisfaction. This implicates that there is no ‘wear out’ of participating to debriefing, in contrast. One may even argue that new users over time become bigger advocates for the debriefing, using the system for this purpose. The type of procedure, years working at the hospital and age did not seem to influence satisfaction, suggesting that there is no extinguish of participation interest and that bias due to the ‘novelty effect’ is minimal [38]. This is an encouraging finding, when implementing innovations in the operating theatre [39, 40].

As to be expected, the primary surgeons, drivers of the initiative, were significantly more satisfied than the participating assisting surgeon, anaesthesiologist and OR nurses in the surgical team. The phenomenon of perceived difference of perception about the same situation between the surgeon and other team members is acknowledged in literature [41–43]. It may also be contributed to the so-called ‘Rashomon’ effect, which occurs when the same events is described in significantly different ways by different people who were involved [44]. Indeed, based on the respective roles, disagreements exist regarding the evidence of events in the OR. Also, subjectivity versus objectivity in perception, memory and reporting is in play, when looking back upon situations. Surgeons, in comparison with the other team members, experience and therefore describe or remember certain events differently. The need for a more multidisciplinary approach to quality improvement initiatives may hence be recommended [37, 45, 46]. Moreover, it is known that communication and the performance of the team is usually graded higher by the surgeon [47–49]. This may further be explained by the fact that this project was an initiative led and strongly supported by the participating surgeons. As participants were asked to voluntarily participate in the TOPPER-trial, it was to be expected that they would be satisfied with the outcomes of project, introducing a positive selection bias in our study. Yet, at the start of the project, only a few anaesthesiologists and nurses felt comfortable enough to decide to participate and sign the informed consent. Interestingly, over time, their participation numbers kept on growing steadily in the study. An effect that can presumably be contributed to the ‘grapevine’, e.g. the positive responses of the already participating team members. Indeed, several healthcare professionals who were initially unsure or even quite sceptical towards the initiative decided to participate in the team debriefing during the trial based on positive experiences shared by their peers. When these second group of adopters overcame their initial scepticism, they reverted their opinion due to actual user experience. They came to better understand how their privacy was protected and experienced the benefits first-hand. As a result, initial laggards became the most important drivers and advocates for the initiative.

In this study, only 3 participants indicated not to recommend participation to peers, of which 1 surgical resident and 2 anaesthesiologists. The surgical resident commented that the answer was ‘no’, because during that particular debriefing, the staff surgeon had to cancel his or her attendance to the team debriefing last minute. Without the staff surgeon, in combination with a relatively ‘uneventful case’, the surgical resident considered the team debriefing to be not so useful. Two anaesthesiologists answered ‘no’ on the question if they would recommend use of the system for team debriefing to peers. Anaesthesiology data were indeed captured in real time by OR Black Box® (e.g. blood pressure, heart rate, oxygenation, etcetera) and reflected in Black Box® output, but the assessment algorithms at that time were not well enough developed to provide the same granularity of assessment as for the surgeons and OR nurses. Also, to protect the privacy of the patient, the OR Black Box® capture of data started when the team started draping, when the patient was hence already under anaesthesia. Recordings were stopped before extubation. Thus, the assumed-to-be more critical moments in anaesthesiology care were not part of the performance report and could not be debriefed using the outcome report. Nevertheless, technical aspects were not the main learning points according to user insights from both surgeons and anaesthesiologists. Take-home-messages, noted during the team debriefing sessions from the anaesthesiologists, were mainly about communication patterns, such as “clear and closed-loop communication is important” and “I should be more specific when asking the surgeon”. In fact, miscommunication has been implicated as one of the major causes of error and adverse outcomes in general surgery [10, 11]. Indeed, these learning aspects need to be taking into account when training surgical teams, which is usually not the case in the separate specialist curricula to date. Authors feel there is an opportunity here for improvement. Apart from training teams in simulative settings jointly, use of the OR Black Box® in team debriefing to look back upon joint performance may help strengthening the surgical safety culture. This, because the OR Black Box® performance report has been built focusing on those aspects regarded to be especially important for joint performance; being human factor skills, like communication and teamwork, next to technical error [50]. Postoperative multidisciplinary debriefing, with the use of the performance report, may hence contribute to prevention of unintentional miscommunication in the OR, especially between the surgeons and anaesthesiologists [51].

Taking into account the different busy work schedules and irregular shifts, planning the team debriefing sessions was difficult sometimes. However, the number of working days between procedure and debriefing session, and number of attending team members did not seem to affect the participant’s satisfaction. Nevertheless, it was decided to reschedule the session, when not enough team members could attend (4 out of 7) to persevere the benefits of multiple viewpoints in the discussion.

Several team members quoted; “because of the Black Box, I was more aware of my communication and this actually improved my way of communicating”. Yet, the performance report showed that there was still some “irrelevant chatting” or “loud music”. This indicates that procedures were performed in the familiar and natural way [26, 52, 53]. Quotes during the debriefings confirmed that there was often a very relaxed and good atmosphere in the OR. This may suggest that surveillance awareness and language did not seem to affect the surgical team’s performance and satisfaction [54].

This is not the first study describing the use of a video and MDR in the operating theatre [55–57]. However, the TOPPER- trial is, to the author’s knowledge, the first study that used a structured and automatically analysed video-assisted performance report as a tool for structured multidisciplinary debriefing, including all members of the operating team. In contrary to others, this study comprehensively explored the participant’s satisfaction with the use of an MDR in the OR, its performance report, and debrief methods. As stated in the literature review by Jue et al*.*, the OR Black Box® is currently the most widespread surgical data recording technology in use in operative settings [57].

This pilot study has some limitations. As mentioned, the participants were asked to voluntarily participate and therefore the results may represent the opinion of beforehand enthusiastic, positively minded participants. One out of the six participating surgeons (MS) was beside a participant, also the project leader. This is an important bias to take into account whilst interpreting the results. To avoid bias, the 6 surgeons did not participate in the data analysis. On the other hand, leading by example is not necessarily wrong in starting disruptive initiatives. One may even argue that such an initiative simply needs a strong driver from within the community and leadership in order to succeed and result in successful implementation. Overall, the level of satisfaction among various users is very high, one may argue that the system lives up to different expectations indeed and certainly did not disappoint.

Another barrier to interpretation of the study may be the fact that participants were asked to speak English during the OR Black Box® recordings. As mentioned above, the data analysis centre is situated in Toronto, Canada, and neither the software nor the ‘raters’ were able to understand and analyse Dutch. To facilitate interpretation of this learning system and maximize the information in the newly designed outcome report, authors chose upfront to revert away from bias that may have been caused by language issues. Indeed, it was believed to be not so much of a problem as the Dutch, especially when highly educated, are fluent in speaking and understanding English [58, 59]. Although it was agreed that during the procedure the team members could always revert back to Dutch if considered necessary, having to speak English was mentioned to be a limitation to the natural workflow in the evaluation of the study, especially by the OR nurses. Another limitation of the study is that its results may have been influenced by the Hawthorne effect, a well-described phenomenon of an unintentional change of behaviour or productivity in response to the presence of an ‘observer’ [60, 61]. It is known that this effect typically fades with time, as the team members are getting used to the observation, especially if the presence of an observer is not directly visible [62]. Our video recordings were made with surveillance cameras that were already mounted into the ceiling in most of our operating rooms. This non-obstructive set-up for observation is likely not to attribute much to a possible Hawthorne effect, as one is likely to forget a camera that is not disturbing one’s activities when focusing at tasks.

The patient itself was not the main subject of this study. Therefore, no correlations could be made with the operative patient outcomes or clinical endpoints. Future studies may prove the direct or indirect benefits for the patients.

Scheduling the multidisciplinary debriefings for such an amount of consecutive surgical cases with so many different team members proved to be a challenge during this study. Authors would have preferred scheduling the debriefings sooner to the surgery, but this proved not feasible in all cases. Nevertheless, having the objective information including the video footage in the outcome report sparked the memory satisfactory, according to participants. Results of this study show that neither the number of team members attending the team debriefing, nor number of workdays between the procedure and debriefing was significantly associated with the satisfaction scores. As a recommendation, authors believe that inviting OR personnel to participate in about 2 multidisciplinary debriefings per year may already be a great facilitator in better understanding each other’s need. Whether or not it is widely generalizable to have an independent person, such as a professor of psychiatry, moderate the sessions and the cost-effectiveness remains open to discussion.

As a result of the positive outcomes of this study, the OR Black Box® system is about to be implemented in full operational modus on multiple clinical operating theatres in our academic medical centre. The performance report is currently, with the help of machine learning software, continuously improving and can now be used for multiple purposes including open surgery in multiple medical centres [63]. Future studies have to determine the effect of including the recording of the entire procedure (start when patient enters the OR and stop when patient leaves the OR) and subsequent anaesthesiology data analysis feedback embedded in the performance report. Further building and incorporating deep-learning artificial intelligence software algorithms capable to process OR Black Box® data are going to provide more accurate assessment of false/true negative/positive events [64]. This may result in scalability of the model, feasibility of team debriefing and an even higher level of user satisfaction. A multicentre study is to be advocated to assess if the OR Black Box® performance report in combination with the Black Box Debrief Model is culturally robust and able to guide discussion during postoperative multidisciplinary debriefings in other medical centres as well.

## Electronic supplementary material

Below is the link to the electronic supplementary material.

Supplementary file1 (DOC 2083 kb)
