# Supplementary material for: Implementing structured team debriefing using a Black Box in the operating room: surveying team satisfaction
Source: Surg Endosc. 2020 Apr 6;35(3):1406–19. doi: 10.1007/s00464-020-07526-3 (PMC7886753; doi:10.1007/s00464-020-07526-3)
Supplement: Supplementary file 1 — Supplementary file1 (DOC 2083 kb) [file 464_2020_7526_MOESM1_ESM.doc]

**APPENDIX**

**Details results factor analysis:**


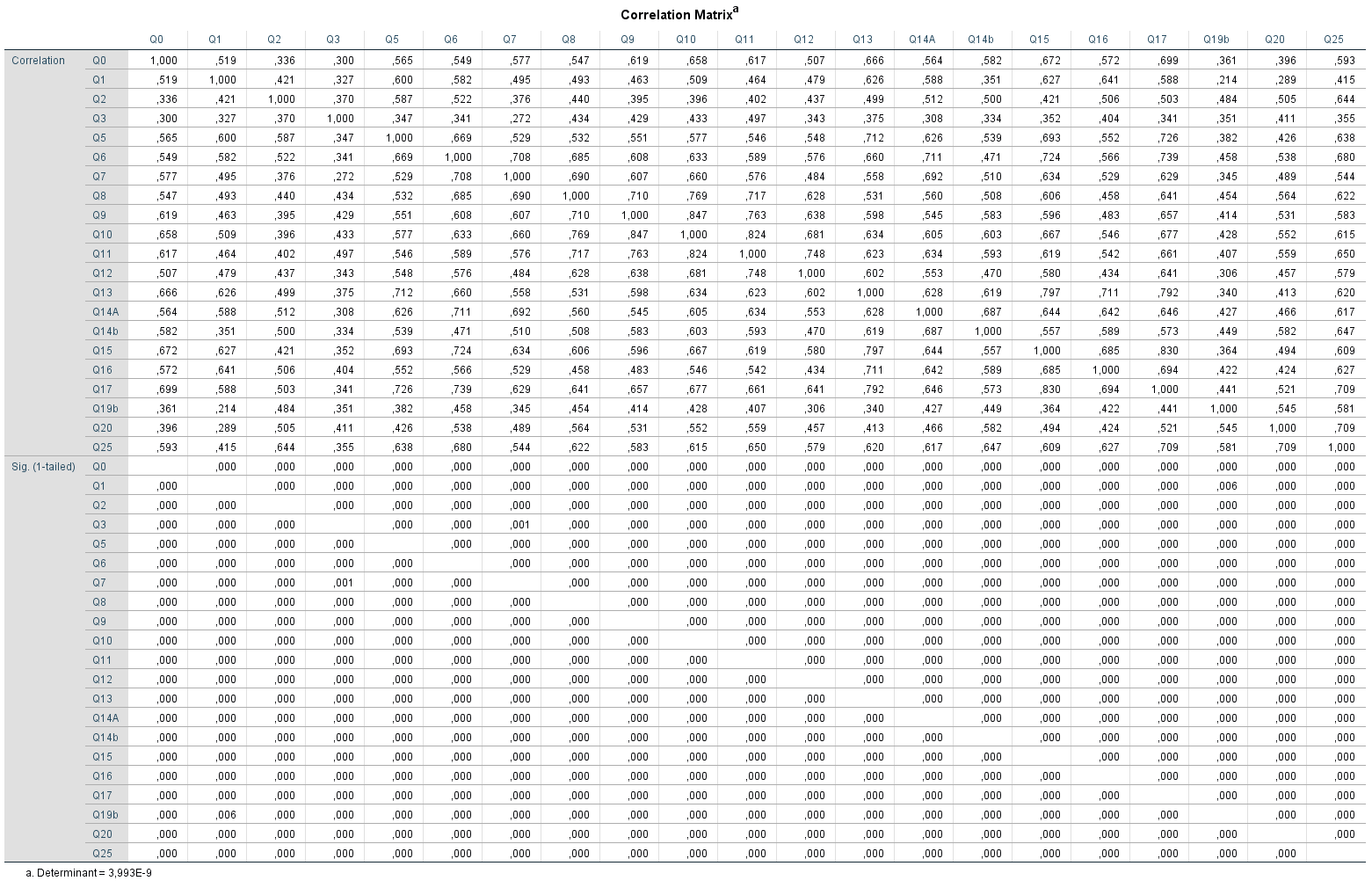


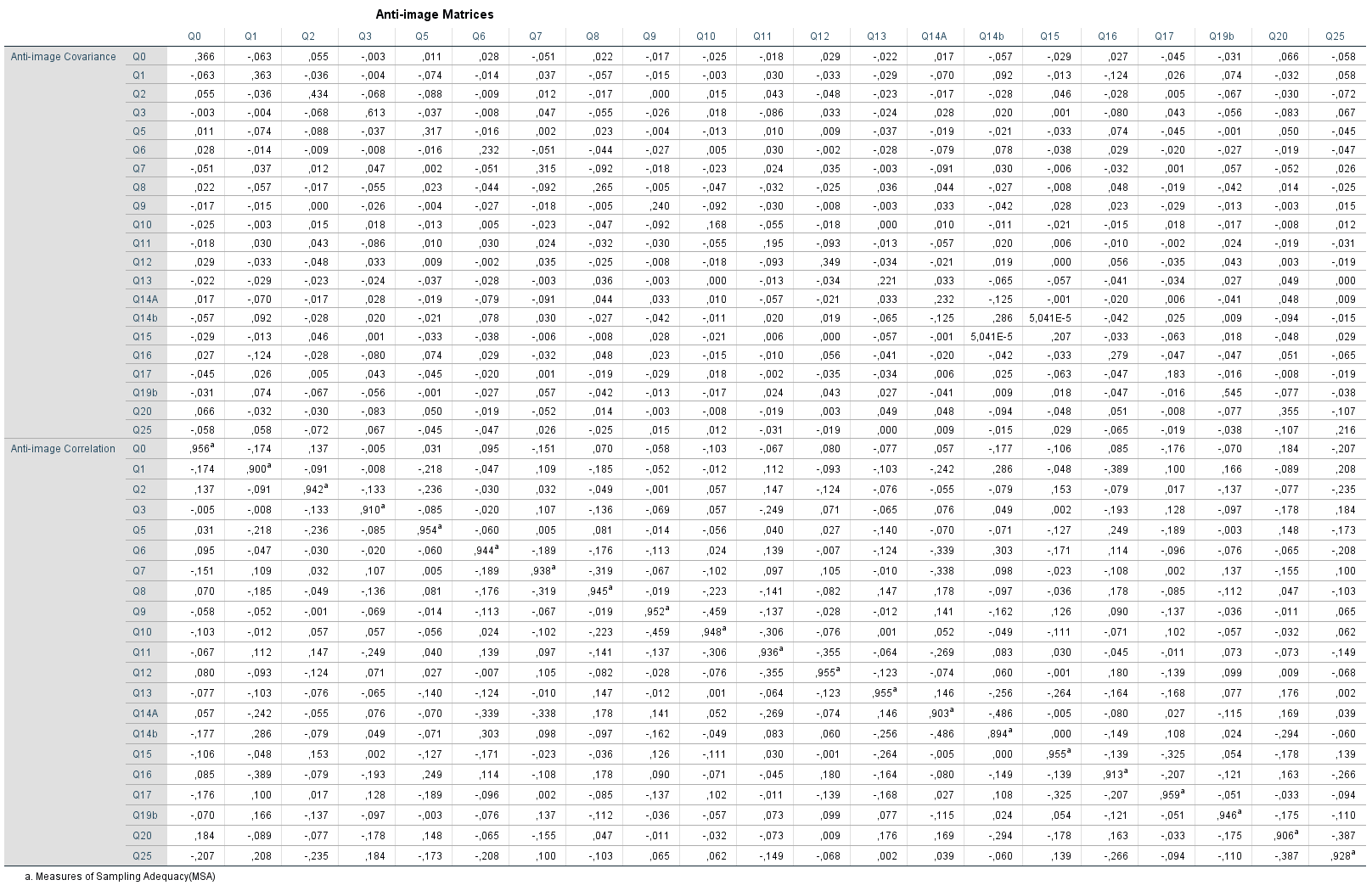


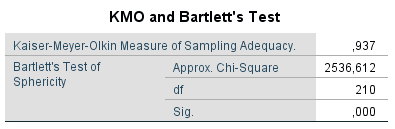


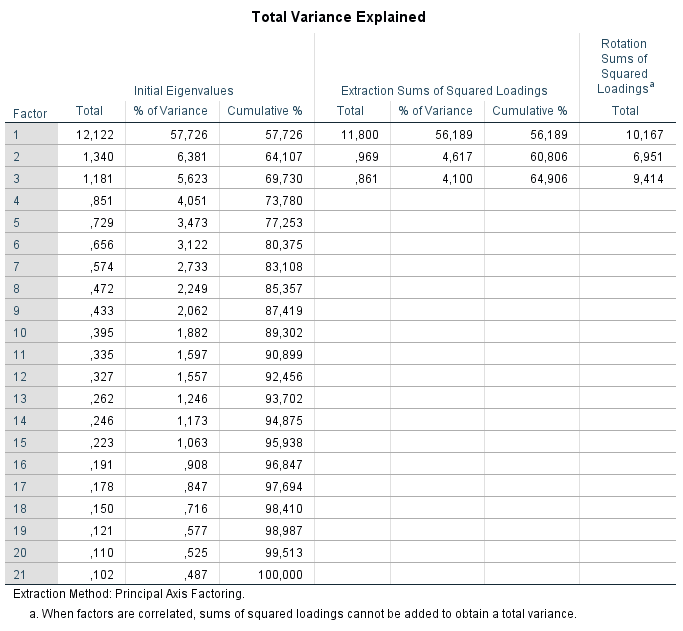


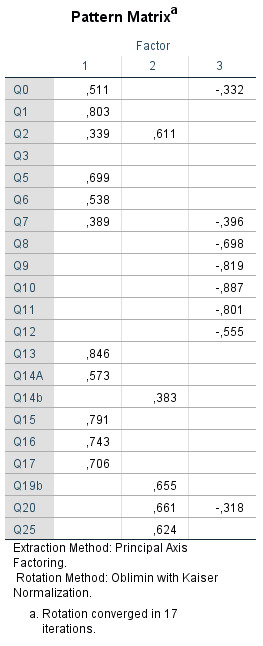


**Original Black Box Satisfaction Questionnaire**

| **TABLE.** Simple linear regression models for the 3 factors. | | | | | | |
| --- | --- | --- | --- | --- | --- | --- |
| **Variables** | **Factor 1** Attitude towards value of team debriefing with the OR Black Box® | | **Factor 2** Satisfaction with the OR Black Box® performance report | | **Factor 3** Attitude towards benefits of team debriefing with the OR Black Box® | |
| Surgical procedure (upper-GI vs adrenal vs colorectal) | *P* = 0.003 | *B** = -0.36 | *P* = 0.40 | *B* =-0.10 | *P* = 0.08 | *B* = 0.22 |
| Role in the OR (ref = main surgeon) assisting surgeon anaesthesiology (including anaesthesia-nurse) OR nurses (SN & CN) | *P* =0.001  *P* = 0.07  *P* =0.99 | *B* = -0.77 *B* = -0.34 *B* = 0.002 | *P* = 0.06  *P* = 0.08  *P* = 0.02 | *B* = -0.43  *B* = -0.31  *B* = -0.42 | *P*<0.0001  *P* = 0.87  *P* = 0.35 | *B* = -0.98  *B* = -0.03 *B* = -0.17 |
| Age | *P* = 0.01 | *B* = 0.02 | *P* = 0.03 | *B* = 0.02 | *P* = 0.01 | *B* = 0.02 |
| Sex | *P* = 0.12 | *B* = -0.28 | *P* = 0.36 | *B* = -0.16 | *P* = 0.40 | *B* = 0.15 |
| Years working at the AMC | *P* = 0.13 | *B* = 0.02 | *P* = 0.63 | *B* = 0.01 | *P* = 0.21 | *B* = -0.02 |
| Number of previously attended Black Box debriefings (first time, 1-5 times, 6-10 times, >10 times) | *P* <0.0001 | *B* = 0.40 | *P* <0.0001 | *B* = 0.54 | *P* <0.0001 | *B* = 0.47 |
| Number of team members attending the debriefing | *P* = 0.43 | *B* = 0.07 | *P* = 0.02 | *B* = -0.19 | *P* = 0.88 | *B* = 0.01 |
| Number of work days between procedure and debriefing | *P* = 0.26 | *B* = 0.01 | *P* = 0.13 | *B* = 0.01 | *P* = 0.78 | *B* = -0.002 |
| **Performance report feedback** | | | | | | |
| Total number of (positive and negative) events in performance report | *P* = 0.004 | *B* = -0.02 | *P* = 0.980 | *B* = 0.00 | *P* = 0.26 | *B* = 0.01 |

***** The beta coefficient is the degree of change in the factor for every 1-unit of change in the predictor variable.
